# Sequential Metabolic Phases as a Means to Optimize Cellular Output in a Constant Environment

**DOI:** 10.1371/journal.pone.0118347

**Published:** 2015-03-18

**Authors:** Aljoscha Palinkas, Sascha Bulik, Alexander Bockmayr, Hermann-Georg Holzhütter

**Affiliations:** 1 FB Mathematik und Informatik, Freie Universität Berlin, Arnimallee 6, 14195 Berlin, Germany; 2 Institute of Biochemistry, University Medicine—Charite, Chariteplatz 1 Sitz: Virchowweg 6, 10117 Berlin, Germany; Karlsruhe Institute of Technology, GERMANY

## Abstract

Temporal changes of gene expression are a well-known regulatory feature of all cells, which is commonly perceived as a strategy to adapt the proteome to varying external conditions. However, temporal (rhythmic and non-rhythmic) changes of gene expression are also observed under virtually constant external conditions. Here we hypothesize that such changes are a means to render the synthesis of the metabolic output more efficient than under conditions of constant gene activities. In order to substantiate this hypothesis, we used a flux-balance model of the cellular metabolism. The total time span spent on the production of a given set of target metabolites was split into a series of shorter time intervals (metabolic phases) during which only selected groups of metabolic genes are active. The related flux distributions were calculated under the constraint that genes can be either active or inactive whereby the amount of protein related to an active gene is only controlled by the number of active genes: the lower the number of active genes the more protein can be allocated to the enzymes carrying non-zero fluxes. This concept of a predominantly protein-limited efficiency of gene expression clearly differs from other concepts resting on the assumption of an optimal gene regulation capable of allocating to all enzymes and transporters just that fraction of protein necessary to prevent rate limitation. Applying this concept to a simplified metabolic network of the central carbon metabolism with glucose or lactate as alternative substrates, we demonstrate that switching between optimally chosen stationary flux modes comprising different sets of active genes allows producing a demanded amount of target metabolites in a significantly shorter time than by a single optimal flux mode at fixed gene activities. Our model-based findings suggest that temporal expression of metabolic genes can be advantageous even under conditions of constant external substrate supply.

## Introduction

The cellular metabolism represents a network of thousands of chemical reactions and transport processes, most of them catalyzed by specific enzymes and transport proteins. The function of this network consists basically in the conversion of chemical compounds that are taken up by the cell from the extracellular space (input) into a large variety of chemical compounds (output) serving either as building blocks for the formation and maintenance of cellular structures (e.g. organelles, cytoskeleton), extracellular structures in case of tissue cells (e.g. glycosaminoglycans, collagens), donors of chemical energy needed to drive otherwise endergonic reactions (e.g. ATP, GTP, CTP, UTP) or signaling molecules released from the cell (e.g. hormones, cytokines, neurotransmitters). As the term ‘biomass’ is commonly reserved to designate all chemical compounds constituting a cell, tissue or organism, we will use the term ‘metabolic output’ to indicate all kinds of chemical compounds required to maintain the structure and physiological functions.

The most important feature of the cellular metabolic network is its ability to adjust the metabolic output to varying external conditions as, for example, an increase in the concentration of pro-inflammatory cytokines or growth factors, depletion of specific substrates (in particular oxygen) or challenges by toxic compounds. This adjustment of the metabolic network to alterations in the external conditions is achieved by selectively increasing or decreasing the capacity of enzymes and membrane transporters. Short-term changes of enzyme activities on a time scale of a few seconds are mainly accomplished by allosteric effects, reversible phosphorylation and de-phosphorylation, and reversible protein-protein or protein-membrane association/dissociation. Long-term changes of enzyme activities on a time scale of minutes, hours and even days result from changes in the expression of enzymes, brought about by changes of the rate of transcription (DNA to mRNA), translation (mRNA to protein) and proteolysis (protein to amino acids).

Long-term regulation of enzyme capacities by temporal gene expression is constrained by the condition that the total protein content of the cell has to be kept within rather narrow bounds (”proteostasis”, i.e., variable allocation of proteins at homeostasis of the total protein pool [1]) of 15–35 percent of cell volume [2, 3]. An upper bound of the protein content is defined through the condition that molecular crowding should not reduce the aqueous space to an extent which impairs the diffusive transport of proteins, metabolites and ions [4]. On the other hand, reducing globally the concentration of metabolic enzymes and membrane transporters lowers metabolic fluxes, as the maximal enzyme capacity is approximately a linear function of the protein abundance. Therefore, regulation of metabolic networks by temporal changes of enzyme expression can be expected to take place under the constraint of a relative constancy of the total cellular protein abundance [2, 5, 6]. As a consequence of this constraint, increasing the abundance of a larger group of enzymes belonging to metabolic pathways that have to be operative under a given extracellular setting, should be accompanied by a decrease of the abundance of enzymes belonging to pathways which are less important or even temporarily dispensable. This principle of “just-in-time” gene expression of metabolic enzymes according to the actual metabolic needs, similar to the just-in-time production as pioneered in the 50s by Toyota in particular, has been validated theoretically and experimentally [2, 3, 7].

Temporal gene expression is generally recognized as an important mechanism with which cells and tissues adapt their metabolism to variations in the external conditions, see e.g. [6, 8]. However, temporary changes in the gene expression of metabolic enzymes and other cellular proteins are also observed under virtually constant external conditions. A prominent example is the so-called “metabolic cycling” with a typical period length of about 300 minutes observed in dense chemostat cultures of budding yeast, *Saccharomyces cerevisiae*, when grown under nutrient-limited conditions [9]. For more than 50 years the cycling in yeast cultures was thought to be directly coupled to the cell division cycle. However, recent studies have provided evidence for the occurrence of cycling in single yeast cells from unsynchronized steady-state (not growing) populations [10, 11]. This finding implies that the cycling is an intrinsic and autonomous feature of yeast cells that is neither dependent on metabolic synchronization of cells, nor an active cell cycle or carbon limitation. Hence, the compelling explanation of metabolic cycling [4] as a means to separate the S-phase of the cell cycle from the oxidative metabolic phase during which an enhanced formation of reactive oxygen species (ROS) through the respiratory chain may occur has to be put into question. Other examples for metabolic cycles are the occurrence of an ultradian metabolic rhythm between oxic and anoxic processes in the diazotrophic cyanobacterium *Cyanothece sp.* ATCC 51142 under constant culture conditions [9], and a self-sustained rhythm in glucose uptake by undifferentiated stem cells that is not coincident with rhythmic expression of clock genes [12].

Given that temporal variations in the expression of metabolic enzymes is a general feature of the cellular metabolism that is not necessarily induced by temporal environmental changes, the question remains what the evolutionary background of such metabolic variations might be. Looking at the evolution of metabolic networks from a Darwinian perspective, one is tempted to figure out the selective advantage that cells existing in a (idealized) constant environment might have acquired by switching between several metabolic states. Here we hypothesize that one possible reason for such metabolic switches is the shortening of the time period to generate a demanded metabolic output with a fixed total amount of protein that can be invested into metabolic enzymes and membrane transporters. The idea underlying our theoretical approach can be illustrated by comparing the metabolic network with a factory that has to deliver a specific quantity of different items (e.g. different types of cars = target metabolites) with a constant number of employees = enzyme protein. One may ask whether it is economically more favorable, i.e., saves total production time, to produce all of these different items all the time in fixed proportions or to use the full man (and machine) power of the factory to produce these items in different proportions over limited time spans. Analogously, we address in this theoretical study the intriguing question whether even without changes of the external conditions (e.g. availability of substrates, strength of hormonal signals etc.) temporal switches in the allocation of protein to the various pathways of the cell’s metabolic network may be advantageous for an efficient biomass production. Importantly, our theoretical approach does not envisage the possibility that the expression of genes can be always optimally tuned in a way that the amount of protein allocated to an enzyme perfectly matches the flux it carries, a principle of gene regulation that has been proposed in [5]. If such an hypothesis is adopted, the metabolic output of the network regulated by a perfect allocation of protein amounts to enzymes and transporters cannot be surpassed by switching between distinct metabolic phases differing by sets of active and inactive genes, which is the framework developed in this paper.

In the first part of the paper, we use a simplistic 3-reaction network to explain our computational concept. In the second part, we provide an application to a more comprehensive metabolic network comprising several pathways of the intermediary carbon metabolism.

## Results

### Modelling approach

A metabolic network is defined by a set of *m* different metabolites *M*_*i*_ (*i* = 1, …, *m*) forming the metabolite vector *M* and *n* different biochemical reactions (including transport processes) carrying the fluxes *v*_*j*_ (*j* = 1, …, *n*), forming the flux vector *v*. We split the metabolite vector into two parts, *M* = M* ⊎ *M* ^#^, where the vector M* comprises the so-called *target metabolites* which have to be produced *de novo*. This production is necessary either to accumulate biomass (this holds specifically in a proliferating cell) or to compensate for the utilization of biomass components due to processes that are not part of the considered network (e.g. ATP consumption by membranous ion pumps). *M* ^#^ is the vector of internal metabolites, i.e., those metabolites which over a sufficiently long time interval are produced and utilized in same proportion and thus do not accumulate or exhaust. Specifically, we will restrict our study to steady-state flux distributions characterized by the condition that at any time all fluxes are constant, such that the concentrations of internal metabolites do not change over time, $\frac{\text{d}M^{\#}}{\text{d}t}=S^{\#}v=0$, and the target metabolites are produced with constant rates, $\frac{\text{d}M^{*}}{\text{d}t}=S^{*}v$. The elements of the stoichiometric matrices *S* ^#^ and S* specify the number of molecules that are utilized and produced in the reactions associated with the internal metabolites and target metabolites, respectively. We study the production (or consumption) of target metabolites over a time interval of length *τ* > 0, which we decompose into a series of *l* (*l* ≥ 1) consecutive shorter time intervals of length *τ*_*k*_ (*k* = 1, …, *l*) whereby $\sum_{k=1}^{l}\tau_{k}=\tau$. The flux distributions *v* ^*k*^ in the various time intervals can be different from each other, but each fulfills the steady-state conditions *S* ^#^ *v* ^*k*^ = 0. The metabolic output of the network produced in the *k*-th interval is given by *M* ^*k*^ ≔ *τ*_*k*_ S* *v* ^*k*^. Throughout this article we will use the term ‘phase’ to denote a time period during which a distinct part of the network is active. Note that some of the target metabolites (e.g. ATP) have to be produced at all times. A reaction *j* that is producing such an indispensable target metabolite thus has to be constrained,

$$\begin{array}{c} v_{j}^{k}\geq mb_{j}, \end{array}$$ (1)

for each flux mode *v* ^*k*^, *k* = 1…, *l* where *mb*_*j*_ gives the flux rate required for maintenance.

Let Γ denote the demanded output of the network, i.e., the amount of target metabolites that have to be produced (or consumed). For example, this can be the amount of nucleotides required for DNA duplication during the S-phase of the cell cycle, or the amount of phospholipids needed to double the surface of all cellular membranes. The aim is to determine flux modes *v* ^*k*^ with intervals lengths *τ*_*k*_, *k* = 1…, *l*, such that the network accomplishes the realization of the demanded total metabolic output, i.e.,

$$\begin{array}{c} \sum_{k=1}^{l}\tau_{k}\;S^{*}v^{k}\geq \Gamma . \end{array}$$ (2)

within the shortest possible time span *τ*.

Obviously, this optimization problem makes only sense if the upper bounds of the fluxes in the network are constrained. The upper bound of a flux *v*_*j*_ is commonly given by *kc*_*j*_ *E*_*j*_, where *kc*_*j*_ is the turnover number of the catalyzing enzyme and *E*_*j*_ its amount. The time-dependent variation of the enzyme amount *E*_*j*_ is the resultant of synthesis and degradation. In a simplified manner this can be expressed through the equation

$$\begin{array}{c} \frac{dE_{j}}{dt}=g_{j}\;ks_{j}\;A\;-\;kd_{j}\;E_{j} \end{array}$$

so that the amount of the *j*-th enzyme at steady-state is given by

$$\begin{array}{c} E_{j}=\frac{g_{j}\;A\;ks_{j}}{kd_{j}} \end{array}$$ (3)

with *g*_*j*_ being a binary variable indicating whether the related gene is active (*g*_*j*_ = 1) or not active (*g*_*j*_ = 0), *A* representing the mass fraction of free amino acids, *ks*_*i*_ representing an overall rate of protein synthesis (including all regulatory steps between transcription and ribosomal translation) and *kd*_*i*_ being the first-order rate constant for the degradation (proteolysis) of the enzyme. Setting the rate of protein synthesis to the product *ks*_*j*_⋅*A* takes into account the fact that the availability of nutrients in general and of amino acids in particular determines the overall rate of protein synthesis [13, 14]. As reasoned above, we make the assumption that a fixed total mass of amino acids is available for the synthesis of the enzymes involved in the metabolic network under consideration:

$$\begin{array}{c} A_{tot}=A\gamma_{A}+\sum_{i}E_{i}\gamma_{i} \end{array}$$ (4)

Here *γ*_*i*_ denotes the molecular mass of the *i*-th enzyme and *γ*_*A*_ is the average molecular weight of one amino acid (126 Da). *A*_*tot*_ is the total mass of free and protein-bound amino acids per gDW, while *A* and *E*_*j*_ are molar amounts also per gDW. Using the relations (3) and (4), it follows that the amount of the j-th enzyme is given by

$$\begin{array}{c} E_{j}=g_{j}\;A_{tot}\;\frac{\eta_{j}}{\gamma_{A}+\sum_{i}g_{i}\gamma_{i}\eta_{i}}, \end{array}$$ (5)

where the parameter *η*_*j*_ ≔ *ks*_*j*_/*kd*_*j*_ controls the amount of protein if the coding gene *g*_*j*_ is active and thus will be referred to as *expression efficiency*. Equation (5) expresses the beneficial effect of a spare protein expression: The more enzymes are switched off (*g*_*j*_ = 0) and the larger the molecular mass (*γ*_*j*_) of these non-expressed enzymes the more protein can be allocated to the active enzymes in the network. With equation (5), the upper bound on the flux rate depending on enzyme *E*_*j*_ is given by

$$\begin{array}{c} v_{j}\;\leq \;ub_{j}≔g_{j}kc_{j}A_{tot}\;\frac{\eta_{j}}{\gamma_{A}+\sum_{i}g_{i}\gamma_{i}\eta_{i}}, \end{array}$$ (6)

flux rates hence given in mol/gDW/h. The lower bounds are defined analogously by *lb*_*j*_ ≔ −*ub*_*j*_, assuming that the turnover number $kc_{j}^{-}$ for the reverse direction has the same value as *kc*_*j*_ for the forward direction of reaction *j*. For irreversible reactions we set $kc_{j}^{-}=0$ and hence *lb*_*j*_ = 0.

#### Constraining the set of simultaneously active genes

In order to determine biologically meaningful solutions, one has to consider that individual genes cannot be arbitrarily activated or inactivated, because certain groups of genes are typically under the control of common transcription factors. In our approach, we adopt the concept of minimal flux modes [15, 16], and introduce groups of collectively regulated genes as *minimal gene sets* (MGS). A MGS is defined as a set of genes coding for a set of enzymes that catalyze the reactions of a stationary flux mode which enables an optimal (according to a chosen objective function) flux through a *single* target reaction. The steady-state flux distribution accomplished by an active MGS is called *minimal flux mode* (MFM). In general, the various target reactions related to the MGSs may either produce an essential metabolite (anabolism) or remove a harmful metabolite (catabolism). In order to assess whether successive switching between different MGSs leads to a decrease of the total production time required for the accomplishment of the metabolic output, we introduce the gain

$$\begin{array}{c} G_{l}≔1-\tau \left(l\right)/\tau \left(1\right), \end{array}$$ (7)

where *τ*(*l*) is the optimal objective value with fixed *l*. Hence *G*_*l*_ gives the gain in time, when *l* different phases can be used instead of only one.

### Illustrating example

**Fig 1:**
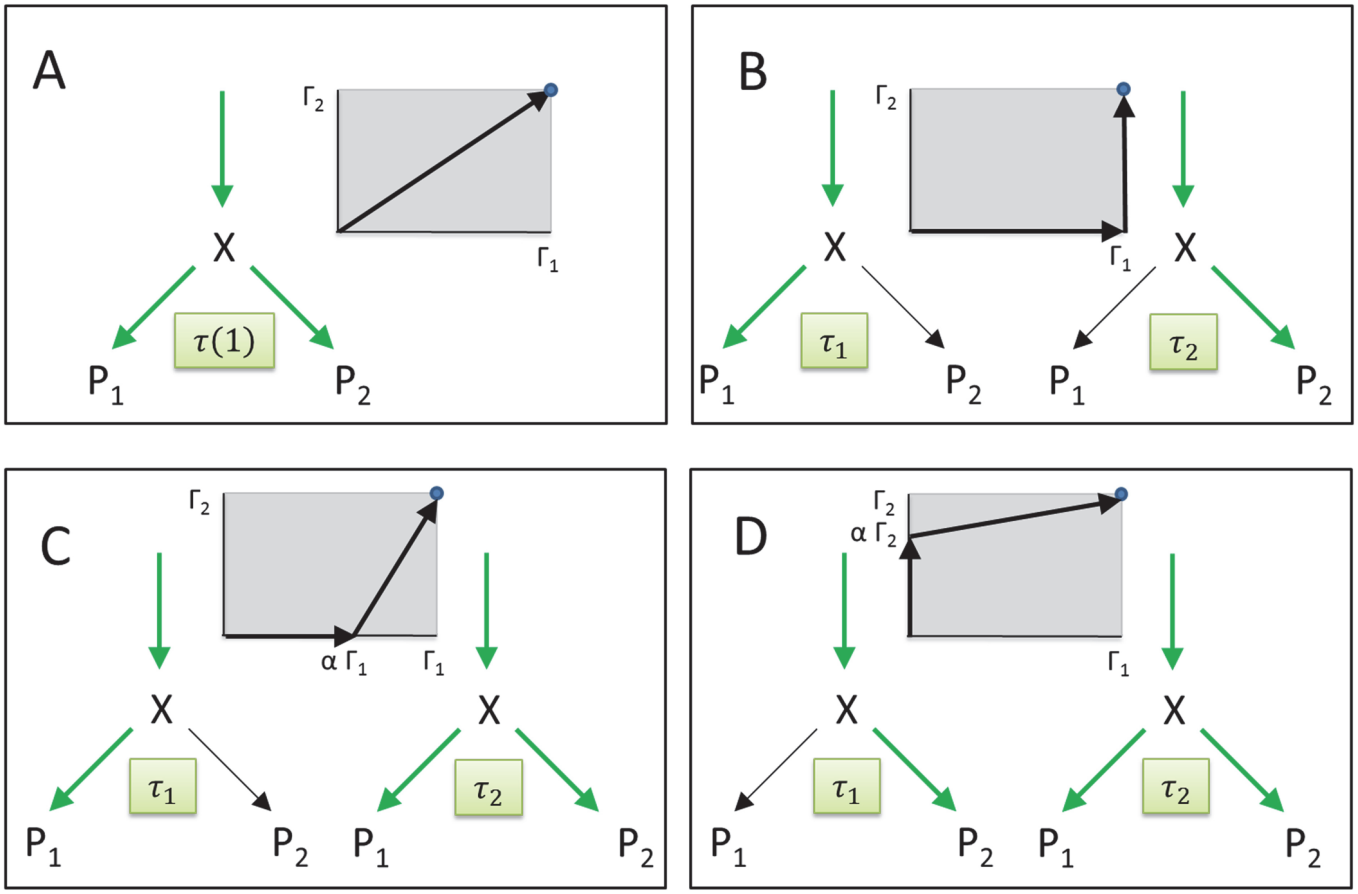
Simplistic metabolic network with two target fluxes. Strategy A: All genes are constantly active, the demanded metabolic output is generated during the time interval *τ*_0_ by a single flux mode composed of the two MinModes *w* ^1^ and *w* ^2^ (= reference case). Strategy B: The two minimal gene sets are separately active, during the first time interval *τ*_1_ only the demanded amount of product *P*_1_ is generated, whereas in the second time interval *τ*_2_ only the demanded amount of *P*_2_ is produced. Strategy C: During the initial time interval *τ*_1_ only the minimal gene set *χ*_1_ is active and only a certain fraction *α* < 1 of the demand for *P*_1_ is produced. Thereafter, the second minimal gene set is additionally activated so that the products *P*_1_ and *P*_2_ are produced simultaneously. Strategy D: During the initial time interval *τ*_1_ only the minimal gene set *χ*_2_ is active, thereafter the second minimal gene set is additionally activated so that the products *P*_1_ and *P*_2_ are produced simultaneously. The gray-shaded panels illustrate the proportions in which the demanded amounts Γ_1_ and Γ_2_ of the two output metabolites are produced in strategies A-D.

We first illustrate our approach for a simplistic metabolic network comprising three irreversible reactions (see Fig. 1). The network has one internal metabolite *X*, one uptake flux *v*_0_, and two target fluxes *v*_1_ and *v*_2_, yielding the target metabolites *P*_1_ and *P*_2_ with demand Γ_1_ resp. Γ_2_. We presuppose two MGSs, *χ*_1_ ≔ {*g*_0_, *g*_1_} and *χ*_2_ ≔ {*g*_0_, *g*_2_}.

The two associated MFMs are *w*_1_ = (*v*_0_, *v*_1_, 0) with *v*_0_, *v*_1_ > 0 and *w*_2_ = (*v*_0_, 0, *v*_2_) with *v*_0_, *v*_2_ > 0. The maximal number of different steady-states (and related phases) required to minimize the production time of the demanded output cannot be larger than the number of different target metabolites (see Methods). Therefore, the maximal number of different metabolic phases for this example is two and we thus have to compare four possible strategies, shown in Fig. 1. Strategy A defines the reference case, i.e., all genes are constantly active. The other strategies assume that during production of the metabolic output the network switches between two phases. Strategy B consists in producing the two relevant products successively: First, the demanded amount Γ_1_ of product *P*_1_ is produced while the pathway for the production of *P*_2_ is switched off. Then, the demanded amount Γ_2_ of product *P*_2_ is produced while the pathway for the production of *P*_1_ is switched off. The switch between these two metabolic phases requires the complete degradation of the enzymes constituting the *P*_1_-synthetizing pathway. As rapidly proliferating cells (without S0-phase of the cell cycle) continuously accumulate biomass during growth without any significant degradation of proteins [17], strategy B should realistically apply to non-proliferating cells. In strategies C and D, during the first metabolic phase time period only one product is produced, and in the second metabolic phase both products are produced simultaneously. Also here switching between the two metabolic phases requires a partial degradation of enzymes of the initially active pathway in order to allocate protein to the second pathway. For this simple system an analytical solution of the optimization problem can be found (see Supplement, S1 Text).

#### Example

With *A*_*tot*_ = 1.8⋅10^6^, equal expression efficiencies (*η*_*j*_ = 1, *j* = 0, 1, 2) and molecular weights (*γ*_*j*_ = 60,000, *j* = 0, 1, 2) and catalytic constants *kc*_0_ = 10 *h* ^−1^, *kc*_1_ = *kc*_2_ = 1 *h* ^−1^, neglecting the additive constant *γ*_*A*_ = 126 in the denominator of equations (5) and (6), the upper boundaries read:

$$\begin{array}{c} \begin{array}{cccccc} \left(A\right) & ub_{0}=100, & ub_{1}=10, & ub_{2}=10 & \;\text{for}\; & v^{1}={\left(v_{0}^{1},v_{1}^{1},v_{2}^{1}\right)}^{⊤}\;\text{(reference}\;\text{case)}\\ \left(B\right) & ub_{0}^{1}=150, & ub_{1}^{1}=15, & ub_{2}^{1}=0 & \;\text{for}\; & w^{1}={\left(w_{0}^{1},w_{1}^{1},0\right)}^{⊤}\\ & ub_{0}^{2}=150, & ub_{1}^{2}=0, & ub_{2}^{2}=15 & \;\text{for}\; & w^{2}={\left(w_{0}^{2},0,w_{2}^{2}\right)}^{⊤} \end{array} \end{array}$$

For the production of Γ_1_ = 10 units of *P*_1_ and Γ_2_ = 50 units of *P*_2_ it needs a production time of *τ* = 10 h in case (A) and only *τ* = 6.67 h in case (B).

Note that for this concrete example, the concept of perfect gene regulation [5] would allow an even shorter production time of *τ* = 2.2 h to be achievable with an optimal single flux mode *v*. Up to scaling by some *α* > 0, it is fixed to *v* = (*v*_0_, *v*_1_, *v*_2_)^⊤^ = *α* ∙ (6, 1, 5)^⊤^ mol/gDW/h (see S1 Text). Perfect gene regulation means that all enzymes work at their maximal capacity, i.e., *ub* = *α* ∙ (6, 1, 5)^⊤^ mol/gDW/h. With the given values for the molecular weights and the catalytic constants this is achieved by choosing the gene expressions *g* ∈ [0, 1]^3^ to be *g*_0_ = 0.12, *g*_1_ = 0.2 and *g*_2_ = 1. The resulting upper bounds are then *ub* = (*ub*_0_, *ub*_1_, *ub*_2_)^⊤^ = 4.545 ∙ (6, 1, 5)^⊤^ = *v*.

For Γ_1_ + Γ_2_ = 100 units, the optimal solution with strategy B always needs *τ*_1_ + *τ*_2_ = 6.67 h, whereas the time needed with a single flux mode (strategy A) varies between *τ*(1) = 5 h in the case Γ_1_ = Γ_2_ = 50 units to *τ*(1) = 9 h for Γ_1_ = 90 units, Γ_2_ = 10 units. When we fix Γ_1_ = Γ_2_ = 50 units and vary instead the catalytic constants *kc*_1_, *kc*_2_, strategy B becomes preferable when the constants are sufficiently different. For example, with *kc*_1_ = 0.5 h^−1^ and *kc*_2_ = 5 h^−1^ we get for strategy A *ub*_1_ = 5 h^−1^ and *ub*_2_ = 50 h^−1^ and therefore *τ*(1) = 10 h. For strategy B the bounds are reciprocally increased by the factor 3/2 or zero and they allow production of Γ in a time of *τ*_1_ + *τ*_2_ = 6.67 h + 0.67 h = 7.3 h only.

#### Empirical analysis

**Fig 2:**
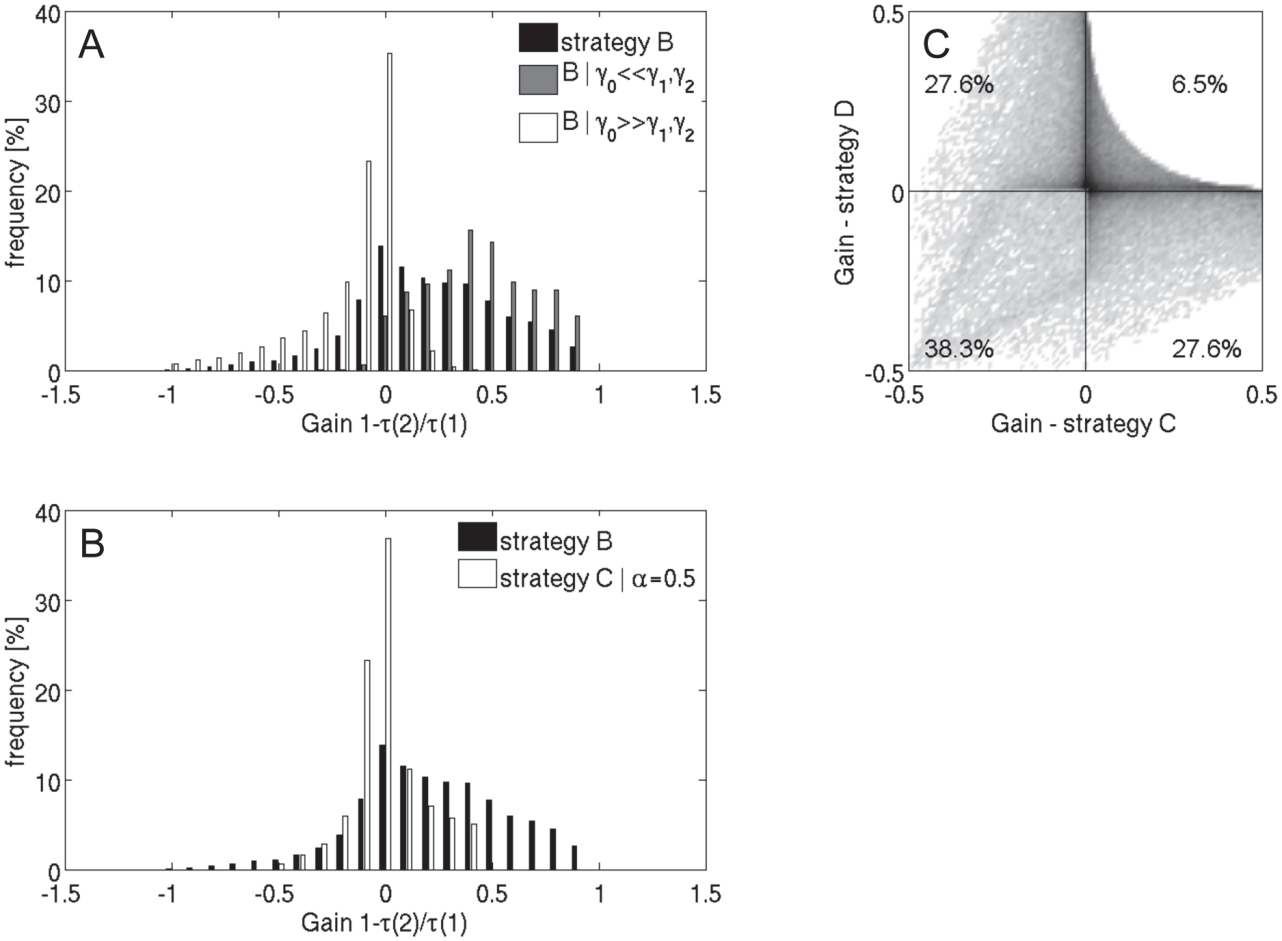
Frequency distribution of the gain achievable with strategies B or C relative to strategy A. The computation of the gain *G*_1_ = 1 − *τ*(2)/*τ*(1) was carried out for 20000 trials, where the turnover number, molecular mass and expression efficiencies of the three enzymes were randomly varied in the range [10^−1^; 10], while the free amino acids were neglected by setting *γ*_*A*_ = 0. A) Strategy B, black bars, all parameters randomly varied; strategy B, white bars, relative molar masses set to: *γ*_0_ = 100, *γ*_1_ = *γ*_2_ = 1; strategy B, gray bars, relative molar masses set to: *γ*_0_ = 1, *γ*_1_ = *γ*_2_ = 100 B) Strategy B, black bars, all parameters randomly varied; strategy C, white bars, all parameters randomly varied and *α* = 0.5. C) Density plot of gain of strategy C versus the gain of strategy D.

The minimal time span required to produce a prescribed amount Γ_1_ of product *P*_1_ and Γ_2_ of product *P*_2_ in the four different gene activation strategies depends on the turnover number, molecular mass and expression efficiencies of the catalyzing enzymes. The minimal production times were computed by randomly varying the numerical values of these parameters in the range from 0.1 to 10 (and in the range [0.01, 100], see S1 Fig.). The relatively small amount of free amino acids was neglected by setting *γ*_*A*_ = 0. For the two strategies, the distribution of the values for the gain function (7) is shown in Fig. 2.

For strategy B, in about 82% of all trials the gain was larger than zero, i.e., the production time was shorter than in the reference case (see Fig. 2 A black bars). We performed a statistical analysis of all trials to figure out those parameter constellations for which a switch between two different metabolic phases is not favorable. This analysis revealed that the success of the strategy to shorten the production time is basically determined by the relationship between the molecular masses and the turnover numbers of the enzymes. The larger the amount of protein allocated to the upstream pathway (*v*_0_), which is mandatory active, compared with the amount of protein allocated to the enzymes of the two downstream pathways (*v*_1_ and *v*_2_) between which can be switched, the lower the benefit of strategy B (Fig. 2 A white bars). In the extreme cases simulated here (*γ*_0_ = 100*γ*_1_, *γ*_1_ = *γ*_2_), strategy B is not favorable for even 55% of the simulated cases. Conversely, if a significantly larger portion of available protein is spent on the downstream operating enzymes, strategy B enables a reduction of the production time in virtually all cases (Fig. 2 A gray bars).

The strategies C and D combine strategy B (exclusive production of only one product) with strategy A (simultaneous production of both products). For fixed *α* they are on average less efficient than strategy B, but nevertheless yielded a reduction of the production time in a substantial number of cases. When we fix the share of Γ_1_ produced in the two intervals to be equal, strategy C has a shorter production time with 66% of the parameter samples. Strategy D shows the same distribution of gain values as strategy C due to the symmetry of the example network. However, it depends on the actual parameter set whether strategy C or D is more beneficial (see Fig. 2 C). Generally, a large benefit of one strategy renders the other strategy less beneficial. There are no parameter combinations allowing for high gains of strategy C and D at the same time. Naturally, there are parameter combinations causing high negative gains in both strategies. Only for 6.5% of the sampled parameter sets, strategies C and D were both favorable. This implies that for a small number of parameter sets the success of one strategy (C or D) entails a failure of the other strategy, because full independency of the parameters sets should result in 11.6% cases (= 34% of 34%) where strategies C and D are both favorable. In summary, despite its simplicity this example points to the important and plausible fact that selective usage of different gene sets and related metabolic steady states should be of specific advantage in cases where the temporary shut-down of certain pathways saves a significant amount of amino acids that can be spent on other pathways to improve their capacity. This principle finding is in line with the results of a recent theoretical study demonstrating that protein abundance of pathway enzymes influences the timing of their activation [18].

### Minimizing the biomass production time in a model of the central carbon metabolism

#### Definition of the network and metabolic objectives

**Fig 3:**
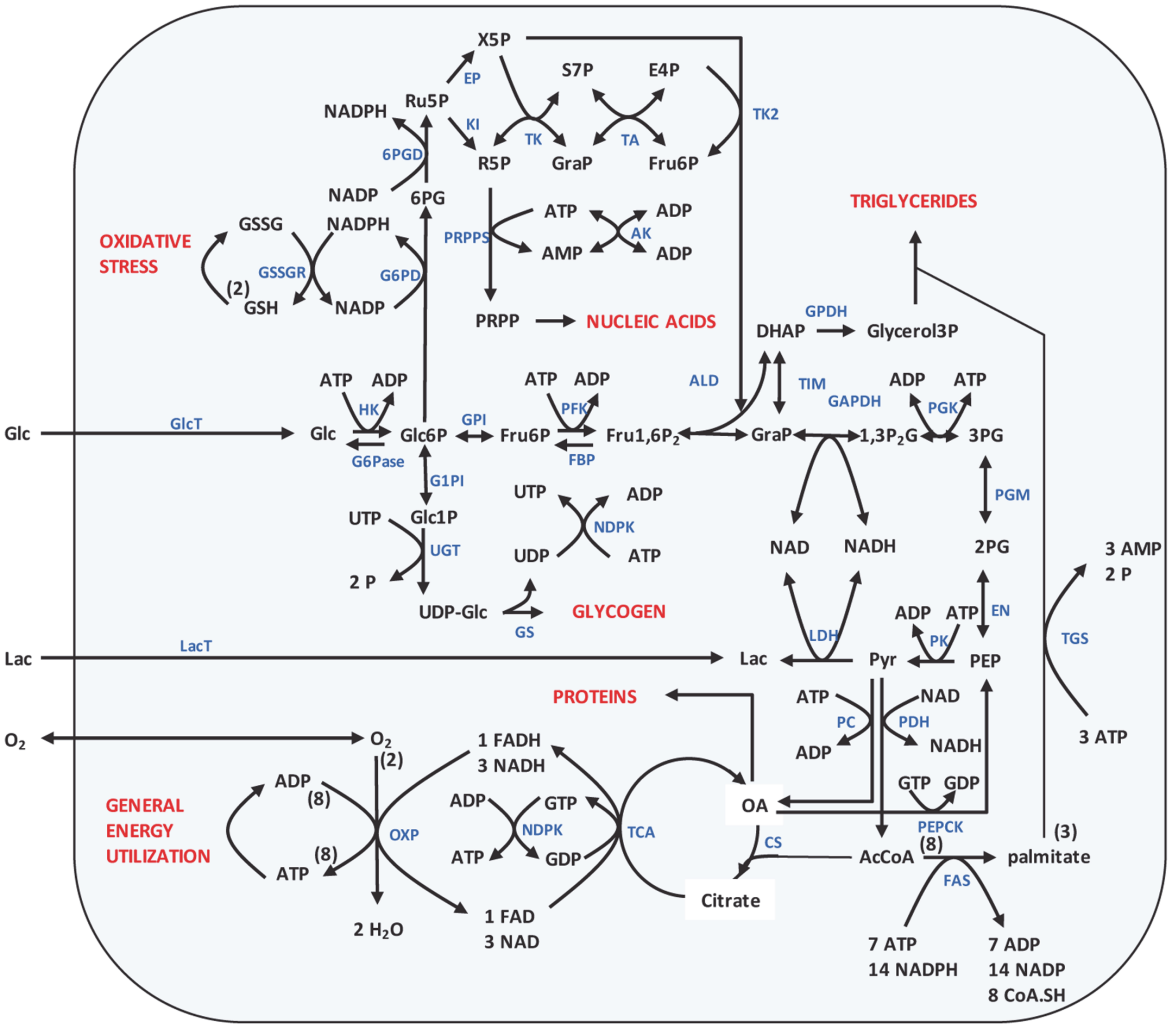
Scheme of the network of the central carbon metabolism, with glucose or lactate as substrates. The metabolic output, i.e., production of triglycerides, nucleic acids, proteins and glycogen are shown in red as well as the permanent energy consumption (ATPase) and oxidative stress (GSHox). Names of all reactions can be looked up in supplemental S1 Table. The metabolites are Lactate (Lac), Glucose (Glc), Glucose-6-*P* (Glc6P), Fructose-6-*P* (Fru6P), Fructose-1, 6-bisphosphate (Fru1, 6P2), Glucose-1-*P* (Glc1P), UDP-Glucose (UDP-Glc), Dihydroxyacetone phosphate (DHAP), Glyceraldehyde phosphate (GraP), 1, 3-Bisphosphoglycerate (1, 3P2G), 3-Phosphoglycerate (3PG), 2-Phosphoglycerate (2PG), Phosphoenolpyruvate (PEP), Pyruvate (Pyr), Oxalacetate (OA), Acetyl-Coenzym-A (ACoA), Gluconate-6-*P* (6PG), Ribulose-5-*P* (Ru5P), Xylulose-5-*P* (X5P), Ribose-5-*P* (R5P), Sedoheptulose-7-*P* (S7P), Erythrose-4-*P* (E4P) and Phosphoribosyl pyrophosphate (PRPP).

Next we investigated a physiologically more meaningful example and applied the proposed optimization approach to minimize the production rate of biomass components in a simplified metabolic network of the cellular carbon metabolism. The reaction scheme of this network is shown in Fig. 3. The network comprises as main metabolic pathways glycogenesis, glycolysis and gluconeogenesis, the pentose phosphate cycle composed of the oxidative and non-oxidative branch, the synthesis of triglycerides and the oxidative energy metabolism. The citric acid cycle, the respiratory chain and the synthesis of free fatty acids and triglycerides are only represented by lumped overall reactions. The considered final output of the network is the production of four macromolecules which are central for maintaining the integrity of the cell and which in dividing cells have additionally to accumulate in the growth phase (G1 of the cell cycle) before cell division: Synthesis of glycogen (an important carbohydrate store), nucleic acids (RNA + DNA), triglycerides (an important energy store), and proteins. The cellular network can exchange oxygen and the metabolites glucose and lactate with the environment. The uptake rate of O_2_ is not constrained; the rate of the membrane transporters for glucose and lactate is subject to the same constraints as all other enzymatic reactions. As already stated in the introduction, some of the metabolic objectives of a network have to be permanently fulfilled during the whole life cycle of a cell, and thus cannot be temporarily switched off. For the exemplary network in Fig. 3, this pertains to the metabolites GSH and ATP. The anti-oxidant GSH protects the cell from reactive radicals and has to be continuously replenished from GSSG. Furthermore, besides the ATP consuming processes utilized by reactions that explicitly occur in the network, a certain fraction of ATP is continuously utilized (termed +ATP utilization in Table 1) to maintain essential cellular processes as, for example, active membrane transport or cell motion. Table 1 quantifies for an average human cell type the demanded output and the brutto reactions relating the metabolites produced in the network to the output of macromolecules. Note that the prescribed fluxes through the target reactions GSSG reduction and surplus ATP production convert into the quantities of ATP and GSH that have to be obligatorily produced in any time interval (see Eqn. 1). The optimization problem (𝒫_1_) (see Methods) to minimize the production time for the four target metabolites glycogen, protein, nucleic acids and lipids thus has to be solved by taking into account the possible consumption of the two alternative substrates glucose and lactate and the two indispensable target (maintenance) reactions ATP consumption and GSSG reduction (see Table 1).

**Table 1:** Demand of output metabolites and associated energy consumption.

Metabolic output of the reaction network		
Target metabolite	biomass reaction for producing 1 unit of target molecule	demanded units
Glycogen	50,000 UDP-Glc → 1 glycogen + 50,000 UDP	5.6 ∙ 10^−6^ mmol/gDW ^1)^
Triglycerides	3 palmitate + 1 glycerol-3-P + 3 ATP → 1 triglyceride + 3 AMP	0.2 mmol/gDW ^2)^
Proteins	400 OA + 1600 ATP → 1 protein + 400 AMP + 1200 ADP	0.016 mmol/gDW ^3)^
Nucleic acids	RNA: 3000 PRPP + 21.000 ATP → 1 RNA + 21.000 ADP	4.48 ∙ 10^−5^ mmol/gDW ^4)^
	DNA: 6 ∙ 10^9^ PRPP + 42 ∙ 10^9^ ATP → 1 DNA +42 ∙ 10^9^ ADP	5.3 ∙ 10^−13^ mmol/gDW ^4)^
GSSG reduction	1GSSG + NADPH → 2 GSH + NADP	0.002 mmol/gDW/h ^5)^
+ATP utilization	ATP → ADP	5 mmol/gDW/h ^6)^

1. Average number of glucose moieties in a glycogen molecule = 50,000. Average MW of glycogen = 70*μ*g/mg protein [19].

2. 0.167 g/gDW lipid [20]. Average MW per triglyceride = 176 + 42 *n* D with n = length of fatty acids. With n = 16 (palamitate) MW = 848 D.

3. 0.78 g/gDW protein [20]. Average size of protein = 400 amino acids. Average MW per amino acid = 126 D. As amino acids are not included into the network model the metabolite oxaloacetate (OA) involved in the transamination of many amino acids is used here as a place holder, i.e., the consumption of amino acids for protein synthesis equals the consumption of OA.

4. DNA: 0.0103 g/gDW [20]. Length DNA (double strand) = 6 ∙ 10^9^ nucleotides. Average MW of single nucleotide = 325 D. RNA: 0.0437 g/gDW [20]. Average length RNA = 3000 nucleotides.

5. GSSG reduction rate in erythrocytes representing a cell type with a high oxidative load [21].

6. Value chosen such that 40% of total ATP utilization is spent on active membrane e processes (predominantly Na-K-ATPase).

### Determination of minimal gene sets

For each of the two substrates glucose or lactate, we computed 6 minimal gene sets (MSGs), each defining an optimal flux mode (MFMs) that produces the maximal amount of one of the four biomass components and the two maintenance metabolites ATP and GSH (see Table 1). Operating at a minimal protein cost implies that spending the total available amount of amino acids to this minimal flux mode maximizes the production rate of the target metabolite. To these 2 × 6 = 12 MGSs we added another group of 8 MGSs which maximize the flux through the four target reactions yielding glycogen, protein, nucleic acids and lipids while maintaining the indispensable target fluxes, i.e., *v*_*j*_ ≥ *mb*_*j*_ for *j* ∈ {GSHox, ATPase}. These 20 MGSs (see S2 Table for the corresponding MFMs) were used to constrain the simultaneous activation and inactivation of genes when solving the optimization problem.

### Specification of turnover rates and molecular masses

Numerical values for the turnover rates *kc*_*j*_ and molecular weights *γ*_*j*_ were taken from the BRENDA data base (http://www.brenda-enzymes.org/). Transport rates for glucose and lactate were taken from [22, 23], respectively. For the overall reaction ‘citric acid cycle’, we took the turnover number of the rate limiting enzyme isocitrate dehydrogenase. The fatty acid synthesis, FS, was assigned a turnover number of 43 *s* ^−1^, according to [24] and oxidative phosphorylation was assigned a turnover rate of 80 *s* ^−1^, which is the minimum of the values for the individual reactions, as retrieved from BRENDA. For Glucose-6-P-dehydrogenase we assigned a turnover rate of 14 *s* ^−1^ according to [25]. The molecular masses assigned to lumped reactions were taken as the sum of the molecular masses of the involved individual enzymes. The numerical values for the catalytic constants and molecular masses used in our calculations are listed in S1 Table. Furthermore we assume that the few unknown turnover numbers are not rate limiting for the network, the respective fluxes are unbounded.

### Specification of the reference case (no switching)

**Fig 4:**
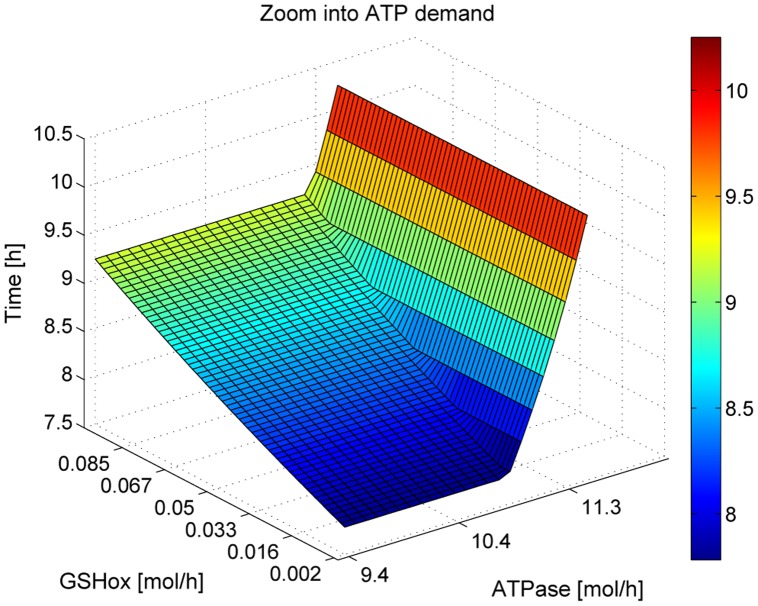
Dependence of the minimal biomass production time in the base condition (all genes active) on the magnitude of the flux through the maintenance reactions. The fluxes through the maintenance reactions were increased. GSHox flux was increased up to 50-fold and ATPase flux 4-fold of their normal values. The surface starts at the bottom with the minimal production time of 8h with all genes active and maintenance demand of 0.002, 5 mmol/gDW/h, see Table 1. Only if ATP consumption by the maintenance reactions is increased by a factor ≥ 2.5, the minimal production time is prolonged. This is due to the fact that fulfillment of the metabolic objectives requires ATP production in all metabolic phases. As long as the responsible reactions are not rate limiting, i.e., their catalyzing enzymes do not operate at the upper flux bound, the rate of ATP synthesis can be increased to balance the additional ATP demand of the maintenance reactions up to an increase to the 2.5-fold of the normal. Below this threshold, only GSSG reduction acts as a bottleneck for biomass production. With 15 mmol/gDW/h of ATP consumption the time for production becomes 13.1 h and if the consumption rate tends towards 18.7 mmol/gDW/h, the total available amount of amino acids has to be allocated to the ATP-producing flux mode, and *de novo* production of biomass is not possible anymore. In contrast, owing to its smallness, variations of the flux through the GSH oxidase reaction have only little impact on the minimal production time. An even 5-fold higher rate of GSH oxidation prolongs the minimal production time by only 0.06 h.

To define the reference case where the network operates with a single stationary flux mode, in which all genes are constantly active and thus all enzymes are constantly expressed, the value of the total mass of available amino acids, *A*_*tot*_, was chosen such that the minimal time for the production of the demanded metabolic output given in Table 2 was 8 hours. A value of 8h for the biomass duplication time lies between doubling times reported for yeast cells (1.25–2 hours) and cancer cells in culture derived from metastatic tumors (∼ 24 hours). Slightly different values of *A*_*tot*_ were obtained depending on the availability of the substrates glucose and lactate (see Table 2). The critical amount of amino acids $A_{tot}^{crit}$ just sufficient to produce the flux through the two maintenance reactions (GSHox and ATPase) without generation of the four biomass components is about $A_{tot}^{crit}=0.023$ mg/gDW, i.e., 20% to 27% of the total available amino acids pool has to be spent on pathways enabling these two permanently active maintenance reactions (see Table 2). Of importance, the flux through the maintenance reactions may even increase under challenging conditions, e.g. if the cell is exposed to a higher osmotic pressure that necessitates the activation of membranous ATP-depending ion pumps to preserve the osmotic equilibrium, or at a higher load of reactive oxygen species (peroxides) enhancing the utilization of GSH. In such situations, at fixed value of *A*_*tot*_, an increase of the flux through the maintenance reactions above the normal values results in a delayed production of the biomass components (see Fig. 4). The times needed for biomass production decrease monotonously with increasing *A*_*tot*_.

**Table 2:** Minimal total molecular mass of amino acids.

substrate	$A_{tot}^{crit}$ in mg/gDW	*A*_*tot*_ in mg/gDW
glc and lac	0.023	0.087
glc	0.023	0.115
lac	0.024	0.087

Minimal total molecular mass of amino acids *A*_*tot*_ required to produce the metabolic output within 8h at different modes of substrate supply. *A*_*crit*_ represents the minimal total molecular mass of amino acids required for only accomplishing the maintenance reactions. All genes were set to be active in these computations.

### Minimizing biomass production time by consecutive switches between active gene sets

Having defined the reference case, we solved the minimization problem (𝒫_1_) (see Methods) to check whether switching between different sets of active genes may significantly reduce the minimal production of the metabolic output. In these computations, the numerical values of the expression efficiencies were put to unity for all enzymes, i.e., the abundance of enzymes according to expression (6) is only controlled by the number and molecular masses of active genes. An optimal solution of the minimization problem does not require more phases than there are different metabolic objectives. Thus we fixed the maximal number of phases where different sets of genes are active to *l* = 4 and solved the optimization problem for an increasing number of phases, *l* = 1, 2, 3, 4. Note that the case *l* = 1 is not identical with the reference case. Solving (𝒫_1_) with *l* = 1 allows inactivating parts of the network, whereas all genes are active in the reference case. As a result the times *τ*(1) are below 8h, see Table 3.

**Table 3:** Minimal production times in 1, 2, 3 and 4 different phases.

Switching MinModes On and Off									
substrates	*l*	*τ*(*l*) [h]	*τ*_1_ [h]	*τ*_2_ [h]	*τ*_3_ [h]	*τ*_4_ [h]	# gene switches	# active genes	
glc, lac	1	7.344					0	41	(*i*)
	2	5.394	2.815	2.580			10	33, 43	(*ii*)
	3	4.788	2.434	0.177	2.177		28 (14+14)	31, 41, 35	(*iii*)
	4	4.774	2.451	2.090	0.099	0.134	31 (12+10+9)	31, 35, 37, 40	(*iv*)
glc	1	7.513					0	41	(*v*)
	2	5.330	2.113	3.217			15	38, 33	(*vi*)
	3	4.812	3.217	1.439	0.157		15 (9+6)	33, 32, 38	(*vii*)
	4	4.794	0.095	0.057	1.425	3.217	17 (6+2+9)	36, 34, 32, 33	(*viii*)
lac	1	7.344					0	41	(*ix*)
	2	5.929	0.706	5.222			6	41, 35	(*x*)
	3	5.857	5.222	0.202	0.433		8 (2+6)	35, 37, 39	(*xi*)
	4	5.848	3.052	2.161	0.202	0.433	10 (2+2+6)	33, 35, 37, 39	(*xii*)

**Fig 5:**
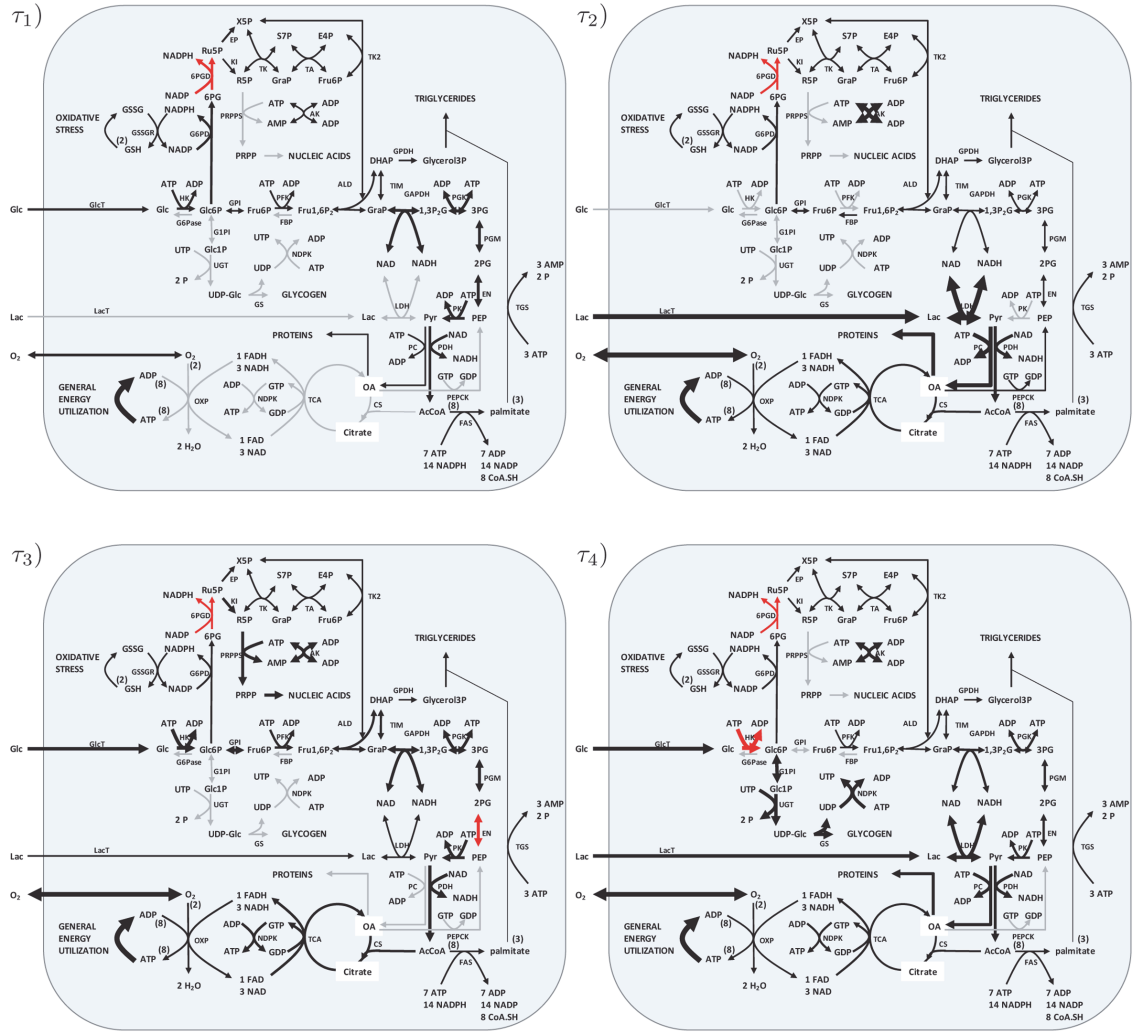
Steady-state flux distributions related to the active gene sets within the four different phases of biomass production. The shown flux distributions correspond to the optimal solution (*iv*) in Table 3. The line width of the arrows corresponds to the flux rate, inactivated reactions are drawn in gray. For the identification of rate limiting reactions, those fluxes which attain their upper bounds are marked in red.

MGS can be switched On and Off from one phase to the next. Times of optimal solutions with 1, 2, 3 and 4 phases. The flux mode of solution (*iv*) is visualized in the network in Fig. 5.

**Fig 6:**
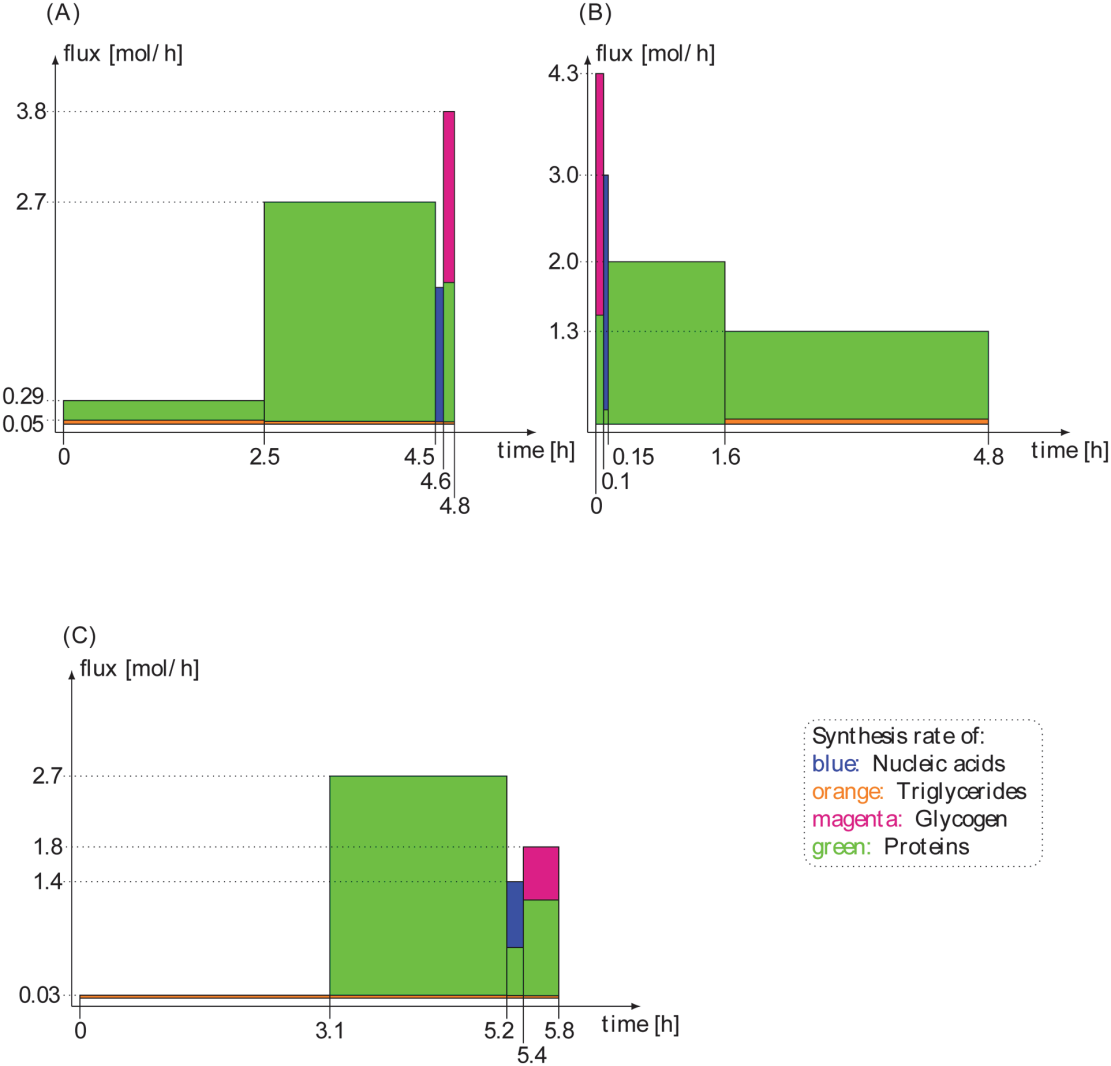
Flux rates through the biomass producing target reactions within various phases for the solution of the optimization problem with *l* = 4 different phases. The size of the colored areas correspond to the amount of biomass component produced in the respective time interval. (A) Glucose and lactate are available substrates, (B) Glucose is the only substrate, (C) Lactate is the only substrate. For the dividing cell, where we assume that genes are only progressively activated (Tab. 4), the resulting production profiles are very similar (not shown).

The minimal production times obtained when the number of possible phases was increased stepwise from *l* = 2 to *l* = 4 are depicted in Table 3 (see Fig. 5 and supplement S2 Fig. and S3 Fig. for the visualization of the *l* = 4 optimal solutions). Intriguingly, the most significant drop of the minimal production time was already obtained by allowing two phases, i.e., switching the network once between two different steady states. A larger number of phases resulted only in a marginal further improvement. The best solution with a minimal production time *τ* = 4.78 h was obtained with three switches between *l* = 4 phases and glucose and lactate as allowed substrates. The relative proportions of the biomass components produced within the four phases and the corresponding flux modes are shown in Fig. 6 and Fig. 5. Although the sequence of phases is arbitrary, we ordered them such that the amount of protein that has to be newly synthesized in the transitions between the different activity states in consecutive time intervals becomes minimal. This choice was motivated by the consideration that both the degradation of proteins and the *de novo* synthesis of mRNA and proteins consumes energy and other metabolic resources. Since the whole protein pool is distributed in every phase, degradation and synthesis are balanced and the cost for degradation is implicitly contained in the synthesis costs.

According to our solutions of the optimization problem, the rate of synthesis of the four biomass components should vary in different phases. In particular, the synthesis of glycogen and nucleic acids (dominated by the *de novo* synthesis of DNA) is predicted to occur only in short phases, whereas the production of the more abundant components (lipids and proteins) occurs in more than one phase. The optimal solutions depend critically on the availability of substrates. If, for example, only glucose is available, the total time interval during which the genes related to protein synthesis are active is longer than in a situation where both glucose and lactate can be used (see Fig. 6 A, B). Interestingly, if the two substrates glucose and lactate are both available, they are used differently within the four phases. During the synthesis of nucleic acids and glycogen, both substrates are used in parallel. In the last phase, where the majority of protein is synthesized, lactate serves as the only substrate.

The quantities of biomass output in the four flux modes with glucose and lactate as substrates are depicted in Fig. 6. Due to the high demand for protein synthesis and the high cost of fatty acid synthesis, the solutions are dominated by these requirements.

### Minimizing biomass production time by successive activation of genes

The optimal solutions in the preceding section were obtained by allowing genes and the related enzymes to be switched on and off in different metabolic phases. This is an unlikely situation in rapidly dividing cells, which run quickly through the G1-phase of the cell cycle without a notable degradation of proteins [17]. To account for this situation, we repeated the calculations under the additional constraint that genes can only be progressively turned on, i.e., that the number of active genes increases monotonously in time. Note that the constraint of a constant total protein pool requires that also in this scenario, a partial degradation of enzymes of the preceding phase has to take place, in order to make amino acids available for the synthesis of the additionally activated enzymes in the next phase. The results are listed in Table 4. The gain, i.e., the relative reduction of biomass production times was only marginally lower than in the preceding section, where gene sets could also be temporarily switched off in subsequent phases. Intriguingly, although the additional constraint that genes can only be turned on results logically in a smaller total number of gene switches (see Tables 3 and 4) the predicted four phases of biomass production are very similar to those obtained if genes are allowed to be alternatingly turned on and off.

**Table 4:** Minimal production times if genes can only be switched on.

Switching MinModes only On									
substrates	*l*	*τ*(*l*) [h]	*τ*_1_ [h]	*τ*_2_ [h]	*τ*_3_ [h]	*τ*_4_ [h]	# gene switches	# active genes	
glc, lac	1	7.344					0	41	(*i*)
	2	5.394	2.580	2.815			10	43, 33	(*ii*)
	3	4.850	2.053	0.180	2.618		18 (6+12)	37, 43, 31	(*iii*)
	4	4.847	2.006	0.089	0.131	2.621	18 (2+4+12)	37, 39, 43, 31	(*iv*)
glc	1	7.513					0	41	(*v*)
	2	5.729	2.512	3.217			10	43, 33	(*vi*)
	3	5.180	1.782	0.181	3.217		16 (6+10)	37, 43, 33	(*vii*)
	4	5.170	0.086	0.086	1.780	3.217	12 (2+6+4)	41, 43, 37, 33	(*viii*)
lac	1	7.344					0	41	(*ix*)
	2	5.929	0.706	5.222			6	41, 35	(*x*)
	3	5.885	0.433	0.230	5.222		8 (2+6)	39, 41, 35	(*xi*)
	4	5.876	0.433	0.230	3.044	2.169	12 (2+8+2)	39, 41, 33, 35	(*xii*)

Times of optimal solutions with 1, 2, 3 and 4 consecutive flux modes. Calculated with the additional constraint that genes can only be turned on, but not deactivated from one interval to the next. Solutions (*iv*), (*viii*) and (*xii*) are visualized in the network in the Supplement, S4 Fig. to S6 Fig.

### Robustness of optimal solutions against random variations of model parameters

**Fig 7:**
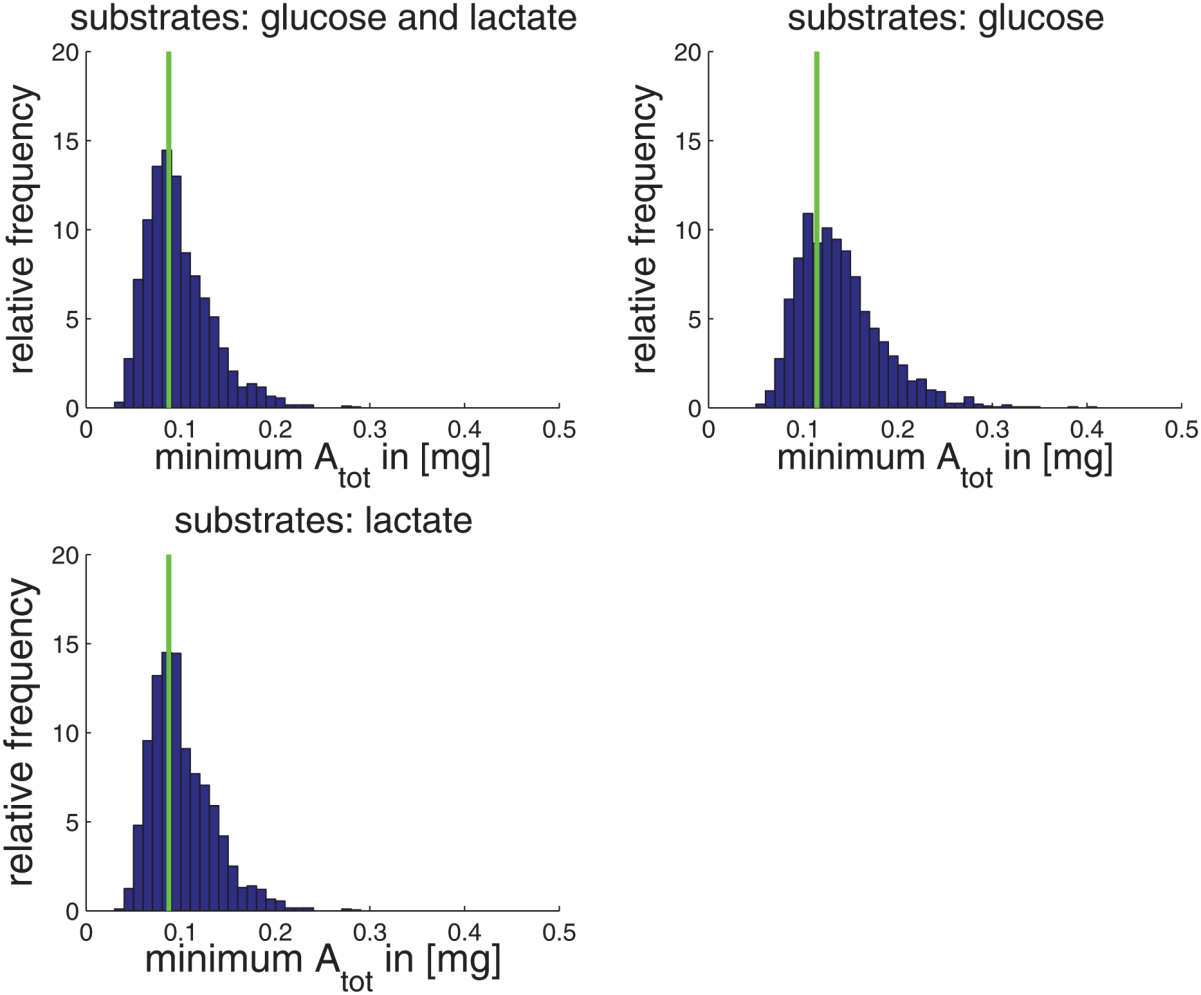
Impact of variations in the enzyme expression efficiencies on the minimal total amino acid mass *A*_*tot*_ required to produce the metabolic output within 8h in the base condition (all genes active, no switch). The frequency distributions are the outcome of 2000 computations of *A*_*tot*_ where the values of the expression efficiencies were sampled from the lognormal distribution *η*_*j*_ ∼ *ln* 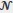
(0, 0.36^2^). The unperturbed values, i.e., *η*_*j*_ = 1 for all reactions *j*, are marked in green.

The computations presented above were carried out under the assumption that the expression efficiencies of all enzymes are equal (*η*_*j*_ = 1 for all *j*). However, expression efficiencies may considerably vary as meanwhile convincingly demonstrated by the generally poor correlation between mRNA and protein levels (see e.g. [26]). Several reasons may account for this observation. The translational efficiency of mRNAs can be controlled by RNA-binding proteins as well as small RNAs, and the half-life of proteins may vary between several minutes and days. Therefore, in order to exclude that the results obtained in the preceding sections critically depend on the choice of the expression efficiencies, we randomly varied their numerical values from a lognormal distribution, i.e., *η*_*j*_ ∼ *ln*𝓝(0, 0.36^2^). As in the preceding sections, we determined for each sample the minimal value of *A*_*tot*_ with which the demanded metabolic output can be accomplished within 8h in the reference state (see Fig. 7). As expected, variations of the expression efficiencies had a large impact on *A*_*tot*_ with deviations of up to a factor of 3 from the unperturbed value *A*_*tot*_ = 0.087 mg/gDW.

**Fig 8:**
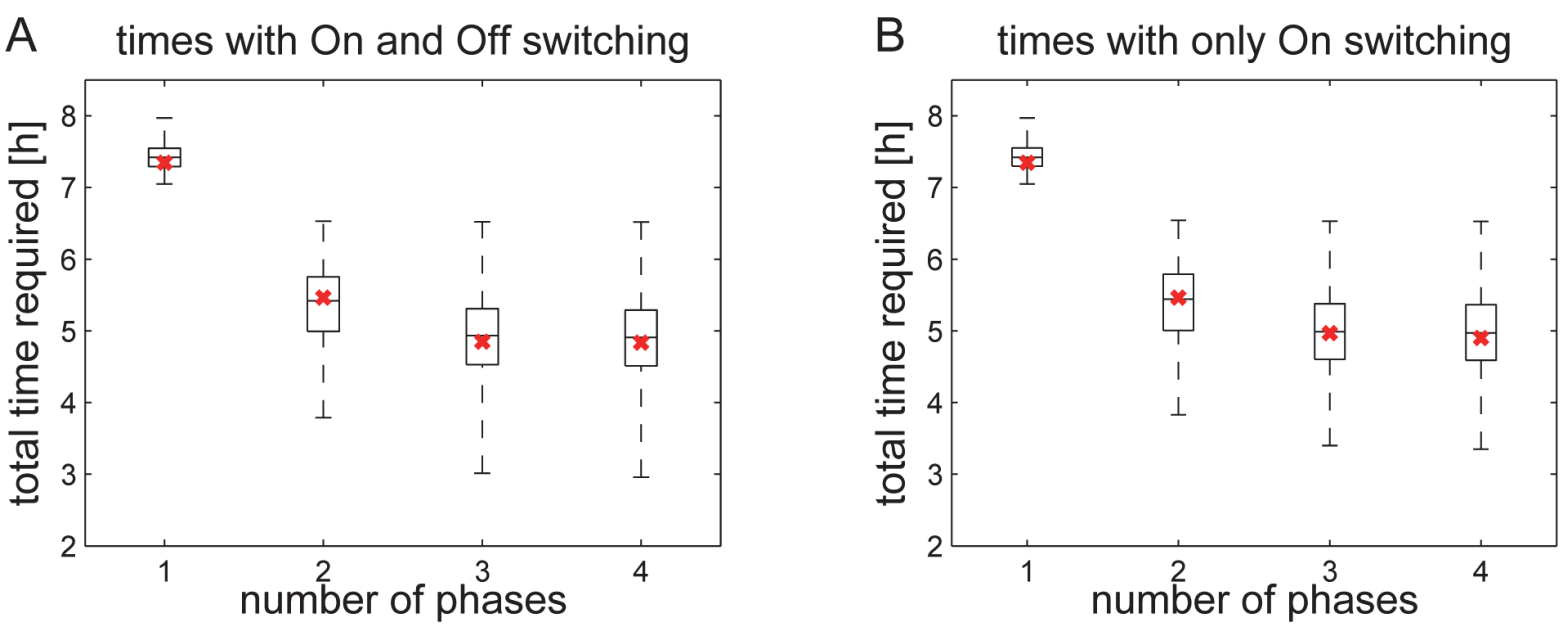
Robustness of optimal solutions. Possible variation ranges of the gain obtained with different numbers of metabolic phases and glucose and lactate as allowed substrates. (A) Genes can be switched on and off, (B) Genes can only be switched on. The red mark represents the base condition (*η*_*j*_ = 1 for *j* = 1, …, *n*).

Then, for each sample, we solved the optimization problem for *l* = 1, 2, 3, 4 different phases and different combinations of glucose and lactate as available substrates. Fig. 8 shows the range of obtained times *τ*(*l*), *l* = 1, 2, 3, 4. Remarkably, for all random samples the strategy of alternating gene switches resulted in a reduction of the total biomass-production-time. Thus, the findings of the preceding section, according to which a variable use of selected gene sets in different metabolic phases allows a reduction of the biomass production time, turn out to be robust against variation of enzyme expression efficiencies.

## Discussion

### General

We have presented a theoretical framework to study the impact of variable usage of genes and related metabolic enzymes on the efficiency of cells to accomplish their metabolic output. The main conclusion of this study is that even in a constant metabolic environment, a temporary switch between different metabolic phases could be a regulatory mechanism that allows a faster and thus more efficient production of the metabolic output. We deliberately emphasize the constancy of the metabolic environment, i.e., the constant availability of nutrients and oxygen, as our approach does not consider that switches between different metabolic states of the cell can be accomplished by other factors such as, for example, hormones, circadian variations of substrate supply, or changes of internal metabolites that upon accumulation or depletion may invoke transcriptional networks. From the evolutionary point of view, exploiting consecutively only certain parts of the whole metabolic network in order to minimize the time required for the production of the metabolic output at limited amount of investable protein could have been one relevant factor that has led to the establishment of temporary gene expression. In this light, temporal gene expression does not only serve as a means to adapt the metabolic output of different cells, tissues and organs to the varying needs of the organism, but also as a strategy to accomplish this output with high efficiency. This view holds in particular for a rapidly dividing cell which may acquire an advantage if its biomass can be reproduced faster than that of its competitors.

### Basic assumptions

One basic assumption made in our approach is that the amount of metabolic enzymes (as well as that of any other protein in the cell) is constrained by the general condition that the total protein mass cannot exceed a certain threshold value without reducing the aqueous phase to an extent that effective communication and catalysis inside the cell gets impaired. This condition was implemented into our approach by rendering the upper bound of fluxes dependent on the number of simultaneously expressed enzymes: The less enzymes are required, and the smaller their molecular masses, the more protein they can acquire. With this constraint on the upper bound of fluxes, the reduction of the biomass production time achieved by the strategy of alternating switches between different metabolic phases appears to be very robust against variations in the expression efficiency of the enzymes in the considered network. Importantly, compared to the approach in [5] also dealing with the implications of molecular crowding for the computation of feasible flux distributions, we deliberately do not allege that the expression efficiency of individual enzymes can be optimized such that an individual enzyme receives just that amount of protein which is necessary to adjust its upper flux bound to the flux it has to carry. A perfect adaptation of the gene expression of metabolic enzymes to flux requirements seems unlikely for several reasons. First, this assumption would entail that all enzymes are saturated, i.e., operate at their maximal capacity (*v*_*max*_). In contrast, all metabolic pathways studied so far have in common that only few enzymes (mostly only a single one) are rate limiting, while the others are not saturated with their ligands, and therefore possess a large overcapacity compared to the actual flux they carry. Second, an investigation of the control of metabolic flux in the model bacterium *Bacillus subtilis* by quantifying fluxes, transcripts and metabolites in eight metabolic states enforced by different environmental conditions revealed that for the majority of enzymes in central metabolism, enzyme concentrations were insufficient to explain the observed fluxes [27]. In line with this finding, a suboptimal control of gene expression was reported to be widespread in bacteria [28]. Third, from an evolutionary point of view, a perfect economical allocation of proteins to the enzymes and transporters of the cellular reaction networks is unlikely to occur, as biological systems that perform multiple tasks face the fundamental trade-off that a given phenotype cannot be optimal at all tasks [29]. Moreover, the capability of cell’s to adapt their proteome to the environmental requirements is restrained by the structure and kinetic properties of the underlying regulatory network [30]. Contemporary gene regulatory networks always bear traces of their evolutionary history: Regulatory circuits as, for example, the operon concept enabling the simultaneous activation and inactivation of genes in functional clusters that may have allowed a perfect adaptation to long-lasting and stable environmental conditions, became highly conserved among different species [31]. Finally, regulation of the cell’s protein abundances is constrained by osmotic and energetic conditions. Maintaining the flux through a reaction while reducing the amount of the catalyzing enzyme unavoidably leads to an increase of the concentration of the reaction substrates—the lower the enzyme capacity, the higher the substrate concentration. This increase of metabolite concentrations may impair the osmotic equilibrium between the cell and its external milieu—a phenomenon that accounts for the impairment of cells in certain types of inborn enzyme deficiencies. The above arguments of course do not deny the existence of a gross relationship between flux strength and enzyme abundances. Taking a more subtle regulation of gene expression into account in our approach would clearly cheapen the gain achievable with metabolic switches. In our calculations of the gain, we neglected the additional costs and the additional time-delay caused by the metabolic switches. Degradation of proteins (mostly by the ubiquitin-proteasome system) and *de novo* protein synthesis consumes some extra ATP. For a rough estimate of the additional costs associated with the *de novo* synthesis of enzymes in various metabolic phases, we added up the mass of protein allocated to those enzymes that are additionally expressed when switching from one phase to the next. This gave an additional ATP demand of 9% resp. 8% for the non-dividing resp. dividing cell, compared with the basal (maintenance) ATP production flux of 5 mmol/gDW/h. Taking into account this extra ATP demand when solving the optimization problem for varying metabolic phases, there was no significant change of the gain in biomass reproduction time. Regarding the time-delay caused by switching from one enzyme expression pattern to the next, experimental observations suggest this switch to occur in a smooth fashion in that the degradation of proteins (and related mRNAs) active in the preceding metabolic phase goes hand in hand with the synthesis of new proteins of the next metabolic phase. Such a tight coupling between protein degradation and synthesis assures the homeostasis of the cellular amino acid pool [32]. The remaining short delay between onset of enhanced proteolysis and onset of *de novo* protein synthesis thus should only marginally prolong the total production time of biomass.

### Metabolic targets

Regarding the choice of metabolic targets for the concrete metabolic network studied in this work, one has to admit that they are not fully independent from each other. For example, a certain basic level of protein synthesis is indispensable at any time for the synthesis of those enzymes required in the current flux state. This, in turn, requires also a basic level of mRNA synthesis at any time. Therefore, the optimal solutions obtained by our approach can only represent rough approximations to the reality just indicating the general trend. Indeed, similar as in our computations, a clear phase shift between the peak activities of the pathways of carbohydrate, lipid, amino acid and nucleotide metabolism has been observed in liver cells [33]. Generally, the conclusion of our study applies to all types of metabolic output as, for example, replacement of damaged cellular building blocks, accumulation of building blocks in growing and dividing cells, or export of metabolites.

### Minimal gene sets

In our approach, the activation and inactivation of genes is accomplished by the regulation of so-called minimal gene sets. These are defined as sets of genes that can only be collectively turned on and off, because they are under the control of common transcription factors. This concept takes into account the existence of signaling pathways and related transcription factors (TFs) that specifically control metabolic pathways of the cell. For example, up-regulation of the transcription factor HIF-1 under hypoxic conditions leads to a higher expression of genes coding for glucose transporters and glycolytic enzymes. Transcription factors belonging to the group of peroxisome proliferator-activated receptors (PPAR) are known to specifically enhance the expression of genes related to lipid storage (PPAR-*γ*) and lipid oxidation (PPAR-*α*). The cellular synthesis of proteins can be globally enhanced by the serine/threonine protein kinase mTOR. Hitherto, the gene regulatory mechanisms underlying the activation of metabolic enzymes by various TFs and the mutual interactions of different TFs at one and the same promotor are not exactly known. Hence, our concept of minimal gene sets appears to be at the moment a reasonable surrogate for the still not completely understood gene regulation of metabolic enzymes. It has to be critically noted, however, that the results of our optimization approach, i.e., the strategy used to sequentially activate metabolic pathways strongly depend on the assumptions made on the cistronic regulation of pathway enzymes [18]. In particular, without constraining the activation of single genes to the activation of commonly regulated gene sets, reduction of biomass production times by alternating metabolic phases becomes even more pronounced.

### Relevance of our findings to the explanation of metabolic cycles

In our computations we have assumed a situation where the cell has to produce a defined metabolic output with minimal production time. Contingent on the simplicity of the chosen exemplary metabolic network, the metabolic output was restricted to some central biomass components as neutral lipids, nucleic acids, proteins and glycogen. In a non-dividing (”resting”) cell, the metabolic output does not lead to an accumulation of the total biomass but instead serves to replenish the permanent loss of biomass components by various degradation and damage processes (lipid oxidation, proteolysis, glycogen consumption, RNA degradation). Let *ε* < 1 denote the fraction of biomass components that is lost in a given time and that needs to be restored within this time period (= homeostasis) to prevent severe cell damage or loss of cell functions. Then the solution of our optimization problem (𝒫_1_) (see Methods) predicts that switching between different metabolic phases permits a faster reproduction of the demand *ε* Γ than achievable with a constant metabolic steady state. This scenario would periodically repeat, thus resulting in a so-called metabolic cycling where each cycle produces only a certain fraction *ε* Γ such that, without permanent loss, after 1/*ε* cycles the complete biomass would have doubled. From this consideration one may conclude that the economic usage of the protein pool that can be allocated to metabolic enzymes is one possible explanation for the periodic switches between distinct metabolic states observed in various non-proliferating cell types as, for example, yeast cells in batch cultures [10, 11]. Independent from external signals, such metabolic cycles can be triggered by cell-autonomous oscillators, i.e., central genes that govern groups of transcription factors and which themselves are periodically varied in their activity by negative feed-back loops, similar as known for the regulation of the central circadian oscillator operating in the neurons of the suprachiasmatic nucleus [34]. In mammals, such oscillators have been found in almost all peripheral cell types [35, 36]. Ex vivo experiments have clearly demonstrated that such cell-intrinsic oscillators may work independently from circadian changes of the cell’s environment (e.g. variations in the concentration of nutrients or hormones) and phases of the cell cycle [37].

## Materials and Methods

### Formulating the optimization problem

To find a sequence of flux modes *v* ^1^, …, *v* ^*l*^ (in mol/gDW/h) that produces all required amounts of metabolic output while maintaining the indispensable target fluxes in all phases, and which minimizes the total time $\tau =\sum_{k=1}^{l}\tau_{k}$ (in h), we solve the following optimization problem:

$$\begin{array}{cccccccc} & \text{Minimize}\;\sum_{k=1}^{l}\tau_{k}\;\text{such}\;\text{that}\; & & & \\ & S^{\#}v^{k}=0,\;\;mb\leq v^{k}, & & & \\ & -\frac{g_{j}\eta_{j}}{\gamma_{A}+\sum_{i}g_{i}\gamma_{i}\eta_{i}}\;kc_{j}^{-}A_{tot}\;\leq \;v_{j}^{k}\;\leq \;\frac{g_{j}\eta_{j}}{\gamma_{A}+\sum_{i}g_{i}\gamma_{i}\eta_{i}}\;kc_{j}A_{tot}, & & & & & & \left({𝒫}_{0}\right)\\ & \sum_{k=1}^{l}\tau_{k}S^{*}v^{k}\geq \Gamma , & & & \\ & \text{with}\;l\in N,\;k=1,\ldots ,l,\;j=1,\cdots ,n,\\ & \text{and}\;\text{variables}\;\tau_{k}\in R_{\geq 0},\;v^{k}\in R^{n},\;g^{k}\in {\left\{0,1\right\}}^{n}. \end{array}$$

#### Including the activation by MGS

We denote the MGS associated with the accomplishment of a flux through the target reaction *s* by the binary vector *χ* ^*s*^, with $\chi_{j}^{s}=0$ if $\chi_{j}^{s}=0$ and $\chi_{j}^{s}=1$ otherwise, where *w* ^*s*^ is the related MFM. In our model, we assume that an arbitrary gene is active if and only if it is member of at least one active MGS. Furthermore, we make the assumption that enzymes coded by inactive genes are also inactive, i.e., the transient phase between switching off genes and degrading the related proteins is not considered. Note also that one gene can be a member of different MGSs. In order to include the additional constraint of collectively activated and inactivated genes into the optimization problem, we introduce binary variables *b*_*s*_, *s* = 1, …, *t*, where *t* is the total number of MFMs, to indicate whether the gene group *χ* ^*s*^ is active or inactive. It follows that *g*_*j*_ = 1 if and only if there exists *s* ∈ {1, …, *t*} with $\chi_{j}^{s}=1$ and *b*_*s*_ = 1. To formulate this as a linear constraint, let $\sigma_{j}=\{s|w_{j}^{s}\neq 0\}$ be the set of MinModes using reaction *j*. Then we have to require that

$$\begin{array}{c} \begin{array}{cccc} g_{j} & \leq & \sum_{s\in \sigma_{j}}b_{s}, & \;\text{for}\;\text{all}\;j=1,\ldots ,n,\;\text{and}\;\\ b_{s} & \leq & g_{j}, & \;\text{for}\;\text{all}\;s=1,\ldots ,t,\;\;\text{and}\;\chi_{j}^{s}=1. \end{array} \end{array}$$

We can now add these constraints to the optimization problem (𝒫_0_). We will call the resulting optimization problem (𝒫_1_), it performs the same optimization, but on a more restricted solution space.

All computations were implemented in Matlab. To solve the optimization problem we used the Gurobi Optimizer 5.6 (http://www.gurobi.com) via Matlab.

The optimization problems (𝒫_0_) and (𝒫_1_) contain as an unknown the number *l* of allowed different metabolic phases. It can be shown that an optimal solution can always be achieved with some *l* bounded by the number of target metabolites. Moreover, the solution of the optimization problem does not depend on the order in which the differently activated MGSs are used, unless further constraints are added evaluating the costs of switching from one phase to the next.

### Upper bound for the number of flux modes in an optimal solution

The number *l* of metabolic phases in (𝒫_0_) resp. (𝒫_1_) is a priori not known. We show now that if the problem is feasible, there always exists an optimal solution using at most as many flux modes *v* ^1^, …, *v* ^*l*^ as there are target metabolites in M*. Hence, in the optimisation problem (𝒫_0_) resp. (𝒫_1_), we can fix *l* = ∣M*∣.

To prove this statement, assume there is an optimal solution consisting of flux modes *v* ^1^, …, *v* ^*l*^ and associated durations $\tau_{1}^{*},\ldots ,\tau_{l}^{*}$ with *l* > ∣M*∣. The constraints $\sum_{k=1}^{l}\tau_{k}S^{*}v^{k}\geq \Gamma$ specifying the metabolic output can be written as (S* *V*)*τ* ≥ Γ, where *V* is the (*n* × *l*)-matrix whose columns are the flux modes *v* ^1^, …, *v* ^*l*^, and *τ* is the column vector (*τ*_1_, …, *τ*_*l*_)^⊤^ (where ∙^⊤^ denotes transposition). Consider the linear optimisation problem $\text{min}\{\sum_{k=1}^{l}\tau_{k}|(S^{*}V)\tau \geq \Gamma ,\tau \geq 0\}$. By definition, $\tau^{*}={\left(\tau_{1}^{*},\ldots ,\tau_{l}^{*}\right)}^{⊤}$ is an optimal solution. From the theory of linear programming (see e.g. [38] Thm. 4.7, p. 121), we know that the corresponding problem in standard form $\text{min}\{\sum_{k=1}^{l}\tau_{k}|(S^{*}V)\tau -\tau^{\prime }=\Gamma ,\tau ,\tau^{\prime }\geq 0\}$ has a so-called basic optimal solution $\bar{\tau },{\bar{\tau }}^{\prime }\geq 0$, for which the number of non-zero components ${\bar{\tau }}_{k}>0$ is at most ∣M*∣ (the number of rows of S* *V*). Such a solution $\bar{\tau }$ describes how to produce the demanded output in minimal time, using at most ∣M*∣ of the flux modes *v* ^1^, …, *v* ^*l*^. To find such an optimal solution, it is enough to solve (𝒫_0_) resp. (𝒫_1_) for the fixed value *l* = ∣M*∣.

## Supporting Information

S1 Fig Frequency distribution of the gain in the minimal example with a wider parameter range. To confirm the independence of the empirical results on the parameter range, the computations for Fig. 2 were repeated, varying the turnover number, molecular mass and expression efficiencies in the range [0.01, 100]. Not surprisingly, the extreme cases of very high gain occur in higher frequency. The conclusions in the main text are not affected by these differences.
(EPS)

S2 Fig Glucose as substrate. The shown flux modes correspond to solution (*viii*) in Tab. 3, see also Fig. 5. (EPS)

S3 Fig Lactate as substrate. The shown flux modes correspond to solution (*xii*) in Tab. 3, see also Fig. 5. (EPS)

S4 Fig Glucose and lactate as substrates. The shown flux modes correspond to solution (*iv*) in Tab. 4. (EPS)

S5 Fig Glucose as substrate. The shown flux modes correspond to solution (*viii*) in Tab. 4. (EPS)

S6 Fig Lactate as substrate. The shown flux modes correspond to solution (*xii*) in Tab. 4. (EPS)

S1 Text Analysis of the minimal example. (PDF)

S1 Table Reaction Names and Parameters. (ODS)

S2 Table MinModes. (ODS)

S1 Files Matlab scripts and model file. (ZIP)
