# Supplementary material for: Sequential Metabolic Phases as a Means to Optimize Cellular Output in a Constant Environment
Source: PLoS One. 2015 Mar 18;10(3):e0118347. doi: 10.1371/journal.pone.0118347 (PMC4365075; doi:10.1371/journal.pone.0118347)
Supplement: S1 Text — (PDF) [file pone.0118347.s007.pdf]

Sequential Metabolic Phases as a Means to Optimize Cellular Output in a Constant Environment

Aljoscha Palinkas, Sascha Bulik, Alexander Bockmayr, Hermann-Georg Holzhütter

Supplementary Information

Analysis of the minimal example

The simplicity of the minimal example allows us to analyze formally how the parameters determine the performance of the different strategies.

Strategy A: Single flux mode For strategy A, the reference case, the demanded output $\Gamma > 0$ is produced by a single flux mode v. Without loss of generality, we assume $\tau v_1 = \Gamma_1, \tau v_2 = \Gamma_2$ and set $r := \Gamma_2/\Gamma_1 = v_2/v_1$. Using the steady-state assumption $v_0 = v_1 + v_2$, we obtain $v^\top = (v_0, v_1, v_2)^\top = v_1 \cdot (1 + r, 1, r)^\top$ (again $\cdot^\top$ denotes transposition). Thus, there is only the unknown v_1, which has to be maximized in order to minimize $\tau(1) = \Gamma_1/v_1$. From $v > 0$, we get $g = (1, 1, 1)^\top$. Setting the kinetic parameters to $\eta_j = 1$, $j = 1, 2, 3$, the upper bounds on the fluxes (cf. 6) are given by

$$ub_j := A_{tot}\, kc_j \,\frac{1}{\gamma_A + g \cdot \gamma} = A_{tot}\, kc_j \,\frac{1}{\gamma_A + \gamma_0 + \gamma_1 + \gamma_2}, \text{ for } j = 0, 1, 2.$$

Maximizing v_1 under the constraint $v \le ub$, we obtain $v_1 = \min(ub_0/(1 + r), ub_1, ub_2/r)$ or equivalently

$$\tau(1) = \frac{\Gamma_1}{v_1} = \max\left(\frac{\Gamma_1 + \Gamma_2}{ub_0}, \frac{\Gamma_1}{ub_1}, \frac{\Gamma_2}{ub_2}\right). \tag{S1}$$

Strategy B: Switching between two MinModes Next we consider the case where the two minimal gene sets χ_1, χ_2 are separately activated in two time intervals with flux vectors w^1, w^2. Here w^1 is only producing the target metabolite P_1 and w^2 only P_2. Applying the steady-state condition, we get $w^1 = (w^1_0, w^1_0, 0)^\top, w^2 = (w^2_0, 0, w^2_0)^\top$. For w^1, we have the upper bounds

$$ub^1_0 := A_{tot}\, kc_0 \,\frac{1}{\gamma_A + \gamma_0 + \gamma_1}, \qquad ub^1_1 := A_{tot}\, kc_1 \,\frac{1}{\gamma_A + \gamma_0 + \gamma_1}, \qquad ub^1_2 = 0,$$

whereas as for w^2 we get

$$ub^2_0 := A_{tot}\, kc_0 \,\frac{1}{\gamma_A + \gamma_0 + \gamma_2}, \qquad ub^2_1 = 0, \qquad ub^2_2 := A_{tot}\, kc_2 \,\frac{1}{\gamma_A + \gamma_0 + \gamma_2}.$$

Maximizing w^1_0 resp. w^2_0 under the constraint $w^1 \le ub^1$ resp. $w^2 \le ub^2$ yields

$$w^1 = \min(ub^1_0, ub^1_1) \begin{pmatrix} 1 \\ 1 \\ 0 \end{pmatrix} \text{ and } w^2 = \min(ub^2_0, ub^2_2) \begin{pmatrix} 1 \\ 0 \\ 1 \end{pmatrix}.$$

For the durations, we get

$$\tau_1 = \frac{\Gamma_1}{\min(ub_0^1, ub_1^1)} = \max\left(\frac{\Gamma_1}{ub_0^1}, \frac{\Gamma_1}{ub_1^1}\right) \text{ and } \tau_2 = \frac{\Gamma_2}{\min(ub_0^2, ub_2^2)} = \max\left(\frac{\Gamma_2}{ub_0^2}, \frac{\Gamma_2}{ub_2^2}\right). \tag{S2}$$

Whether or not the solution w^1 and w^2 outperforms the single flux vector v, i.e., whether or not $\tau_1 + \tau_2 < \tau(1)$ depends on the demand Γ and the upper bounds ub, ub^1, ub^2. We discuss two cases in more detail.

First suppose ub_0 is small, such that $\tau(1) = (\Gamma_1 + \Gamma_2)/ub_0$ and $ub_0 < ub_1^1, ub_2^2$. It follows $\tau_1 < \Gamma_1/ub_0$, $\tau_2 < \Gamma_2/ub_0$ and so $\tau_1 + \tau_2 < \tau(1)$. In other words, switching from w^1 to w^2 is more efficient than the single flux mode v.

Second, assume ub_0 is large, such that $\tau(1) = \Gamma_1/ub_1 \geq \Gamma_2/ub_2$. Using Eqn. **??**, we get $(\Gamma_1 + \Gamma_2)/ub_0 \leq \Gamma_i/ub_i$, which implies $\Gamma_i/ub_0 \leq \Gamma_i/ub_i$, for $i = 1, 2$. Since $ub_0 \geq ub_i \Leftrightarrow ub_0^i \geq ub_i^i$ and using Eqn. **??**, we get $\tau_i = \Gamma_i/ub_i^i$, for $i = 1, 2$. The switching solution thus has the duration $\tau_1 + \tau_2 = \Gamma_1/ub_1^1 + \Gamma_2/ub_2^2$. As long as Γ_2/ub_2^2 is not too small, this will be larger than $\tau(1) = \Gamma_1/ub_1$, the duration of the single mode solution. Taking a closer look at the ratio Γ_1/Γ_2, we observe that a smaller value of Γ_1 and a larger value of Γ_2 in this situation are favorable for the the single mode solution. On the one hand, increasing Γ_1 by a factor $c > 1$ increases also $\tau(1)$ by c, whereas $\tau_1 + \tau_2$ increases by a strictly smaller factor (as long as $\Gamma_2 > 0$). On the other hand, decreasing Γ_2 has no effect on $\tau(1)$, while the duration of the switching solution is decreased. We conclude that the single mode solution performs best compared to the switching solution, i.e., $\tau(1)/(\tau_1 + \tau_2)$ is minimal, if we have equality in our assumption, i.e., $\Gamma_1/ub_1 = \Gamma_2/ub_2$, or equivalently $\Gamma_1/\Gamma_2 = ub_1/ub_2$.

Strategies C or D Between the two extreme strategies A and B, there are the intermediate strategies C and D. Strategy A can be seen as the limit case of strategy C or D, when τ_2 goes to zero. Strategy B is a limit case of C resp. D, when v_1^2 resp v_1^2 vanishes.
